# Change over time in perceived schoolwork pressure and associations with emotional problems among 11–16‐year‐olds: A repeat cross‐sectional study in Wales, UK

**DOI:** 10.1002/jcv2.70005

**Published:** 2025-03-03

**Authors:** Jessica M. Armitage, Gemma Lewis, Nicholas Page, Foteini Tseliou, Rebecca Anthony, Simon Murphy, Graham Moore, Stephan Collishaw

**Affiliations:** ^1^ Wolfson Centre for Young People's Mental Health Cardiff University Cardiff UK; ^2^ Division of Psychological Medicine and Clinical Neurosciences MRC Centre for Neuropsychiatric Genetics and Genomics Cardiff University Cardiff UK; ^3^ Division of Psychiatry Faculty of Brain Sciences University College London London UK; ^4^ Centre for Development, Evaluation, Complexity and Implementation in Public Health Improvement (DECIPHer) Cardiff University Cardiff UK

**Keywords:** adolescence, emotional problems, schoolwork pressure, time trends

## Abstract

**Background:**

Emotional problems among adolescents have increased substantially in recent years, and there is evidence that schoolwork pressures have also increased globally. We examine trends in perceived schoolwork pressure and emotional problems in Wales between 2002 and 2021, and associations between the two over this period.

**Methods:**

Repeat cross‐sectional data were used from surveys conducted in secondary schools in Wales. Participants were 11–16‐year‐olds (*n* = 318,554). Measures included self‐reported perceived schoolwork pressure and emotional problems (HBSC‐SCL).

**Results:**

The percentage of students reporting a lot of perceived schoolwork pressure increased from 21% in 2002, to 24% in 2004. Rates then declined until 2009, dropping to 13%, before increasing to nearly 26% by 2021. Students reporting the most pressure were females in older school years, with up to 57% of females in Year 11 reporting a lot of perceived pressure in 2021. Emotional problems followed a similar trend, decreasing between 2002 and 2009, and then increasing between 2009 and 2021. Between 2009 and 2019, estimates of the cohort difference in emotional problems reduced from 2.23 (95% CI = 2.12, 2.34) to 1.64 (95% CI = 1.54, 1.75) when adjusting for changes in schoolwork pressure. This is consistent with the hypothesis that schoolwork pressure may be driving some of the increase in emotional problems over time.

**Conclusions:**

Our findings showed a decline in perceived school pressure until 2009, followed by a rise until 2021. Increases were particularly apparent among female adolescents, mirroring trends in emotional problems. Although a range of competing causal explanations cannot be ruled out, overall increases in schoolwork pressure from the late 2000s may have contributed to the rise in adolescent emotional problems over this period.

## Graphical abstract

Our findings show an increase in perceived school pressure from 2009 among students in Wales. These increases mirrored trends in emotional problems. Although a range of competing causal explanations cannot be ruled out, overall increases in schoolwork pressure from the late 2000s may have contributed to the rise in adolescent emotional problems over this period.

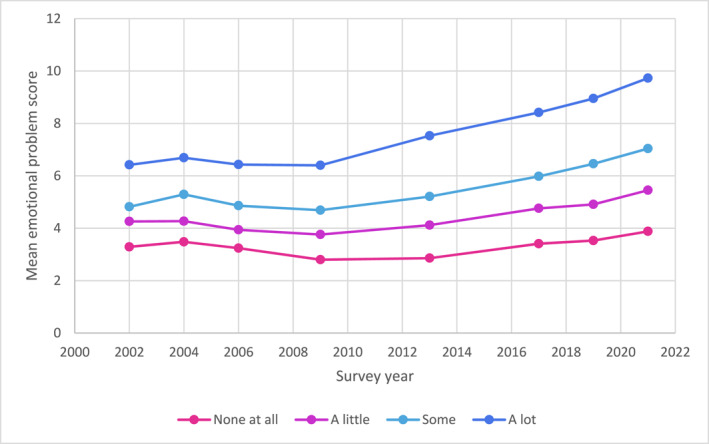

**Key points**

**What's known?**

- Emotional problems among adolescents have increased substantially in recent years, but epidemiological evidence testing reasons for this increase are scarce and an urgent priority.

**What's new?**

- Using repeat cross‐sectional data, we show the percentage of students reporting a lot of perceived schoolwork pressure increased from 13% in 2009 to 26% by 2021. Overall increases in schoolwork pressure from the late 2000s may have contributed to the rise in adolescent emotional problems over this period.

**What's relevant?**

- Understanding why young people experience high levels of pressure at school is a major public health concern, and one that is necessary to informing interventions to support young people cope with pressures, and in shifting academic demands placed on young people. These will be key to preventing further increases in youth mental health problems.

## INTRODUCTION

Improving the mental health of young people is a public health priority, particularly because problems like depression and anxiety are more prevalent now compared to previous generations (Armitage et al., 2023; Sadler et al., 2018). Mental health difficulties can have severe and long‐lasting repercussions, and there is evidence that social, educational and health outcomes are getting worse for more recent generations (Sellers et al., 2019). Understanding reasons for trends in youth mental health is crucial to informing appropriate policy and public health responses to prevent further increases.

Studies testing possible explanations for increasing emotional problems among young people are sparse (Armitage et al., 2024). In the UK, studies have investigated change in substance use, eating behaviours and weight perception (Gage & Patalay, 2021), the role of friendships and bullying (Anthony et al., 2023), and changes in family life (Collishaw et al., 2012, 2019; Schepman et al., 2011). However, only a few studies have directly tested whether accounting for population‐level changes impact estimates of trends in youth emotional problems. These studies paint a mixed picture; one showed that accounting for trends in bullying, cyber‐bullying and friendship quality, made little difference to estimates of the population‐level increase in emotional problems (Anthony et al., 2023). In contrast, evidence suggests that increases in parental mental health problems have contributed, at least in part, to long‐term trends in youth emotional problems (Schepman et al., 2011). Evidence also implies that a rise in family poverty and social inequality in the 21st Century may play an important role in more recent trends (Armitage et al., 2024). In particular, increases in anxiety disorders among young adults have been observed coinciding with the economic crisis and subsequent austerity (Slee et al., 2021). In Wales, trends in emotional problems over time have been shown to be less pronounced for those from medium‐ and high‐affluent backgrounds (Anthony et al., 2023).

Another factor that could be related to the rise in youth mental health problems is schoolwork pressure (Steare et al., 2023). Substantial changes have occurred across schools and society over the last decade, with young people facing increased pressures to pursue higher education (Jaremus et al., 2023; Schoon, 2010). At the same time, austerity measures have forced schools to operate with fewer resources, while increasingly being expected to compensate for challenges such as the rise in youth mental health problems. This has placed increased pressure on schools which have likely increased school‐related stress in turn, on young people (Högberg et al., 2019, 2020).

Early work investigating the role of perceived schoolwork pressure revealed an emerging gender gap with respect to worries about school performance in Scotland (West & Sweeting, 2003). Between 1987 and 1999, the percentage of females who worried ‘a lot’ about doing well in school increased, while the percentage of males worrying decreased. The same study showed increases in psychological distress for females but not males, which were linked to the proximity of data collection to exams in the later survey only. More recently, research in Sweden has shown that schoolwork pressure continues to impact females more than males (Cashman et al., 2023; Högberg et al., 2019), and this explains around half of the difference in the gender gap in psychosomatic symptoms across time (Högberg et al., 2020). In other countries, like the Netherlands, findings have suggested that if schoolwork pressure had not increased, wellbeing may have improved rather than worsened over time (De Looze et al., 2020). Few studies to date have compared trends in schoolwork pressure for individuals of different ages or socioeconomic backgrounds. Doing so is crucial as there has been an increase in socioeconomic inequalities across several domains of adolescent health and mental health over time (Elgar et al., 2015).

The first aim of the present study was to assess change in perceived schoolwork pressure among adolescents in Wales since 2002, both overall and by age, gender, and family affluence. We then investigated trends in emotional problems over the same time period, and compared associations with perceived school pressure to determine whether these varied across time. A final aim was to test the degree to which any changes in perceived schoolwork pressure are related to changes in youth emotional problems. To test these aims, we used repeat cross‐sectional data. Repeat cross‐sectional data involves survey data that is administered to new samples at successive time points. We use data collected from secondary school‐aged children in Wales between 2002 and 2021.

## METHODS

### Study design

Repeat cross‐sectional data were taken from surveys conducted in secondary schools in Wales in 2002, 2004, 2006, 2009, 2013, 2017, 2019 and 2021. Questionnaires prior to and including 2013 were completed as part of the Health Behaviour in School‐aged Children (HBSC) study: a collaborative cross‐national survey of adolescent health and wellbeing, supported by the World Health Organization (https://hbsc.org/). From 2017 onwards, national data were collected via the Student Health and Well‐being survey, led by the Wales‐wide School Health Research Network (SHRN) at Cardiff University (https://www.shrn.org.uk/). The latter survey was developed from the former with HBSC surveys in 2017 and 2021 incorporated into the now larger SHW survey (Murphy et al., 2021).

Data obtained from these surveys constitute nationally representative samples of secondary school pupils. Up until and including 2013, schools were selected using a two‐stage sampling process in which they were first stratified according to local authorities and free‐school meal eligibility, and then randomly selected. Head teachers from each school were contacted through letters and follow‐up phone calls and provided written consent for their school to participate. Selected schools then randomly chose one class (approximately 25 students) in each year group to particpate. All surveys, excluding 2002 and 2006, assessed individuals in every school year (approximate ages 11–16 years). Surveys carried out in 2002 and 2006 did not include students in Years 8 or 10. The current study uses individuals across all school years where available, with sensitivity analyses restricted to those in Years 7, 9 and 11 (see Supporting Information S1).

Trained fieldworkers attended each data collection to ensure sufficient support and participating schools received £150 to cover any costs incurred through participation. Since 2017, surveys have been teacher‐led and delivered by SHRN to all maintained secondary schools in Wales, with no fieldworkers or financial incentives. All surveys post‐2017 were completed during the first school term (September–December), but some earlier surveys, including 2002 and 2006, were completed in the second school term (January–April).

#### School selection and participation

Previous research using these samples has shown that accounting for differences in school selection and participation makes little difference to overall trends (Anthony et al., 2023), therefore all schools were included in all analyses. See Table 1 for information about schools across surveys.

**TABLE 1:** Sample characteristics of 11–16 year olds participating in the Welsh school surveys in our study between 2002 and 2021.

	2002 (*n* = 3442)	2004 (*n* = 6156)	2006 (*n* = 3977)	2009 (*n* = 7544)	2013 (*n* = 8432)	2017 (*n* = 85,795)	2019 (*n* = 99,128)	2021 (*n* = 98,088)
Number of schools surveyed	61	64	70	83	82	193	201	204
Age								
Year 7	1093 (31.8%)	1099 (17.8%)	1248 (31.4%)	1343 (17.8%)	1719 (20.4%)	17,634 (20.6%)	20,786 (21.0%)	19,483 (19.9%)
Year 8	‐	1284 (20.9%)	‐	1624 (21.5%)	1743 (20.7%)	18,274 (21.3%)	20,942 (21.1%)	20,203 (20.6%)
Year 9	1250 (36.3%)	1359 (22.1%)	1434 (36.1%)	1626 (21.6%)	1782 (21.1%)	18,420 (21.5%)	20,712 (20.9%)	21,220 (21.6%)
Year 10	‐	1257 (20.4%)	‐	1479 (19.6%)	1669 (19.8%)	16,589 (19.3%)	19,046 (19.2%)	19,352 (19.7)
Year 11	1090 (31.9%)	1157 (18.8%)	1295 (32.5%)	1472 (19.5%)	1519 (18.0%)	14,878 (17.3%)	17,641 (17.8%)	17,830 (18.2%)
Gender								
Male	1749 (50.8%)	2928 (47.6%)	1917 (48.2%)	3646 (48.3%)	4206 (49.9%)	41,762 (48.1%)	48,553 (48.3%)	50,021 (51.0%)
Female	1693 (49.2%)	3228 (52.4%)	2060 (51.8%)	3898 (51.7%)	4226 (50.1%)	44,033 (50.7%)	50,575 (50.3%)	48,067 (49.0%)
Family affluence								
Low	1073 (31.2%)	1683 (27.3%)	1095 (27.5%)	2793 (37.0%)	2064 (24.5%)	18,182 (21.2%)	21,415 (21.6%)	27,699 (28.2%)
Medium	1469 (42.7%)	2599 (42.2%)	1622 (40.8%)	3142 (41.7%)	3860 (45.8%)	37,880 (44.1%)	42,779 (43.2%)	31,625 (32.3%)
High	900 (26.1%)	1874 (30.5%)	1260 (31.7%)	1609 (21.3%)	2508 (29.7%)	29,733 (34.7%)	34,934 (35.2%)	38,764 (39.5%)

Ethical approvals for surveys carried out in 2013, 2017, 2019 and 2021 were obtained from the Cardiff University School of Social Sciences Research Ethics Committee. Surveys prior to this were delivered by Welsh Government.

### Measures

#### Perceived schoolwork pressure

Schoolwork pressure was assessed across all surveys by asking participants ‘How pressured do you feel by the schoolwork you have to do?’. Responses included ‘Not at all’, ‘A little’, ‘Some’, ‘A lot’. Main analyses investigate this variable as a 4‐point scale, with a higher score denoting greater perceived pressure.

#### Emotional problems

The HBSC Symptom Check List (HBSC‐SCL) is an eight‐item questionnaire that assesses the presence of somatic and psychological symptoms. We focus on the 4‐item psychological subscale which has been previously validated against established measures of depression (Anthony et al., 2023). Participants responded to items related to the frequency in which they had felt each of the following over the past 6 months: (a) Feeling low, (b) Irritability or bad temper, (c) Feeling nervous and (d) Difficulties in getting to sleep. Responses on each item included ‘About every day’, ‘More than once a week’, ‘About every week’, ‘About every month’, ‘Rarely or never’. Responses were summed to form a scale score ranging from 0 to 16, with a higher score indicating higher emotional problems. The internal consistency of the items was adequate in all survey years, ranging from 0.72 to 0.78.

#### Socio‐demographics

Students reported their gender as either boy, girl, refusal to answer, or neither word describes me (the latter response was introduced in 2019). Those in 2019 (*n* = 1472, 1.2%) or 2021 (*n* = 3691, 3.0%) who stated that neither gender best described them were excluded from analyses as temporal change for these groups could not be examined due to the lack of longer‐term data. The same was done for those who refused to answer (see Table S1 for further details).

The Family Affluence Scale comprises indicators of material affluence (Hartley et al., 2016), including the household's number of cars, computers, whether the individual had their own bedroom and whether they had a family holiday abroad in the past year. Surveys from 2013 include two additional items on dishwasher ownership and number of bathrooms. Items available for each survey year were summed and split into three groups denoting low, medium, or high family affluence based on survey year specific centiles.

### Analyses

For descriptive purposes, we compared the percentage of students in each survey reporting ‘a lot’ of schoolwork pressure—for whole samples and then stratified by school year, gender, and family affluence. Linear regressions tested change in mean perceived schoolwork pressure over time with survey year as predictor, and with and without adjustments for age, gender, and family affluence. Survey year was treated as a categorical variable with a reference category of the 2009 survey. This was used after descriptive analyses revealed increases in schoolwork pressure and emotional problems from this survey year onwards. Multi‐level models (MLMs) explored possible variation between the 239 schools included. MLMs with school‐level random intercepts included cohort as an explanatory variable, and were used to test for possible school effects on perceived schoolwork pressure; namely, the degree to which the pupil variance was due to differences between schools. MLMs achieve this by partitioning the total variance into between‐ and within‐school components. A further model then examined whether between school variance differed across surveys. Variation across schools was minimal, therefore further interaction analyses compared differences in schoolwork pressure over time by gender and by family affluence only. All analyses were repeated to examine change over time in emotional problem scores.

Associations between perceived school pressure and emotional problems were examined among all individuals, stratified by survey year, to assess whether these varied across time. Analyses then further stratified by gender, age and family affluence to compare associations among these groups over time. Multi‐level models explored whether accounting for differences between schools had an impact on the association between schoolwork pressure and emotional problems across surveys.

Finally, to test the degree to which any changes in perceived schoolwork pressure may account for changes in youth emotional problems, schoolwork pressure was added as a covariate to models comparing cohort differences in adolescent emotional problems, with differences between unadjusted and adjusted estimates of survey year interpreted as reflecting a possible contribution of changes in school pressure. All statistical analyses were conducted using STATA 17 (StataCorp, 2021).

## RESULTS

### Descriptives

Information relating to perceived schoolwork pressure, emotional problems, age, gender, and family affluence were available across surveys for 312,562 individuals (see Table 1 and Table S1). All surveys had similarly equal samples of males and females and sociodemographic groups, although earlier surveys had fewer groups classified as ‘high affluence’ (Table S2).

### Research question 1: How has schoolwork pressure changed over time in Wales?

**FIGURE 1:**
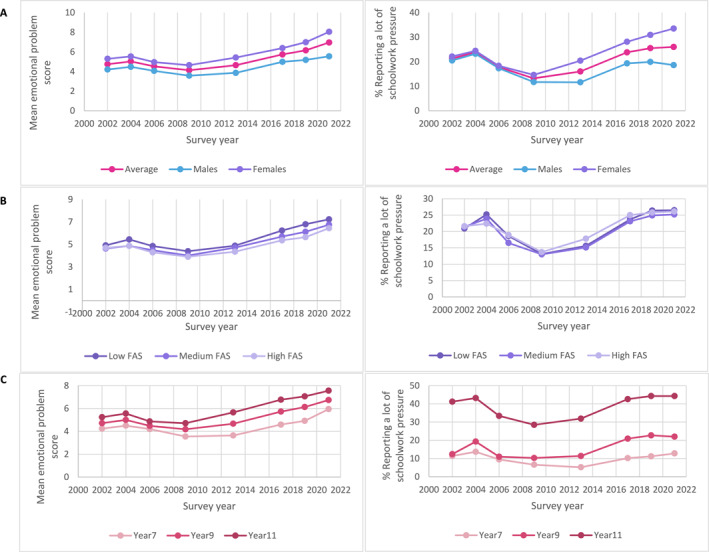
Mean emotional problem scores and percentage of students aged 11–16 reporting ‘a lot’ of schoolwork pressure between 2002 and 2021 by (A) gender, by (B) family affluence, and by (C) age.

The percentage of students reporting a lot of schoolwork pressure increased between 2002 and 2004, and then declined between 2004 and 2009, before showing a substantial increase between 2009 and 2021 (Table S3). Those reporting the most schoolwork pressure tended to be female and in older year groups (Figure 1), with up to 57.4% of females in Year 11 in the most recent survey reporting a lot of pressure (Tables S3 and S4). Interaction analyses confirmed gender differences in trends in school pressure: the increases in perceived schoolwork pressure compared to 2009 observed in 2013, 2017, 2019, 2021, were each more marked for girls than boys (Figure 1 and Table S5).

There were no clear differences in reports of schoolwork pressure among students from high or low family affluence (Figure 1; and see Tables S3 and S4 for breakdown by gender, family affluence, and grade). Increases in school pressure between 2009 and 2013 and 2017 did not markedly vary by family affluence however, there was some indication that families of higher affluence experienced smaller increases in schoolwork pressure between 2009 and 2019, and between 2009 and 2021, although effects were small (Table S5).

Multi‐level models with school‐level random intercepts revealed that 1.5% of the variance in schoolwork pressure can be attributed to differences between schools. Further models revealed that between‐school variance in schoolwork pressure varied across survey years, with variation across schools decreasing over time (Table S6). This is likely due to changes in sample size (pupils and schools) over time.

### Research question 2: How have emotional problems changed over time in Wales?

Emotional problem scores followed a similar trend to schoolwork pressure, with mean increases noted between 2002 and 2004, followed by a reduction up until 2009 (Figure 1 and Table S7). Subsequent surveys showed a large and continuous increase between 2009 and 2021. Mean emotional problem scores increased from 4.13 (95% CI = 4.04, 4.21, SD = 3.76) in 2009 to 6.95 (95% CI 6.93, 6.98, SD = 4.60) in 2021, representing a medium effect size of 0.67. Estimates of change in emotional problems between 2009 and 2021 increased after accounting for differences in the demographic profiles of the surveys (Table 2).

**TABLE 2:** Regression coefficient estimates for change over time in emotional problem score among students in Wales between 2009 and 2021.

	*N*	Main effect of cohort (unadjusted)		Main effect of cohort (adjusted for gender, age, family affluence)		Main effect of cohort (adjusted for gender, age, family affluence, and schoolwork pressure)	
		Estimate (95% CI)	*p* value	Estimate (95% CI)	*p* value	Estimate (95% CI)	*p* value
2009 (ref)	7544						
2013	15,976	0.51 (0.37, 0.66)	<0.001	0.62 (0.47, 0.76)	<0.001	0.55 (0.41, 0.68)	<0.001
2017	93,339	1.49 (1.38, 1.60)	<0.001	1.69 (1.58, 1.79)	<0.001	1.27 (1.16, 1.37)	<0.001
2019	106,672	2.01 (1.89, 2.12)	<0.001	2.23 (2.12, 2.34)	<0.001	1.64 (1.54, 1.75)	<0.001
2021	105,632	2.61 (2.49, 2.73)	<0.001	2.79 (2.68, 2.90)	<0.001	2.12 (2.01, 2.21)	<0.001

*Note*: Estimates use 2009 as a reference. All models adjust for random effects of school.

Females had higher emotional problem scores across all survey years (Figure 1), as did students in Year 11 relative to Years 7 or 9 (Figure 1). Linear regressions investigating interactions between survey year and gender revealed that between 2009 and 2021, increases in emotional problems were more marked among females relative to males (Table S6). Interactions with family affluence suggested that the increase in emotional problems between 2009 and 2017 and between 2009 and 2019 was less marked among families of higher affluence versus those from lower affluent families (Table S8).

**FIGURE 2:**
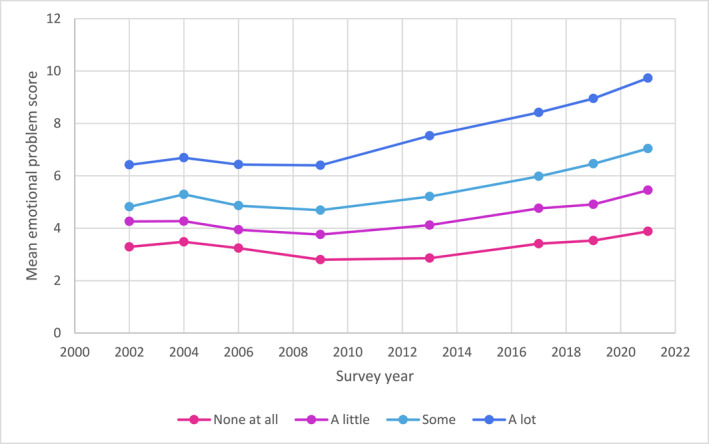
Mean emotional problem scores based on degree of schoolwork pressure between 2002 and 2021.

When mean emotional problem scores were stratified by schoolwork pressure, findings revealed increases across survey years for all adolescents, with increases greatest for those reporting more schoolwork pressure (see Figure 2).

### Research question 3: Have associations between schoolwork pressure and emotional problems changed over time?

Greater perceived schoolwork pressure was associated with greater emotional problems in every survey and for each demographic group, with associations increasing substantially over time (see Models 1–4 in Table 3). Three Multilevel models that accounted for school‐level effects revealed highly consistent results (Table S9).

**TABLE 3:** Regression coefficient estimates for associations between perceived schoolwork pressure and emotional problems among students in Wales between 2002 and 2021.

	2002 (*n* = 3442)	2004 (*n* = 6156)	2006 (*n* = 3977)	2009 (*n* = 7544)	2013 (*n* = 8432)	2017 (*n* = 85,795)	2019 (*n* = 99,128)	2021 (*n* = 98,088)
	Estimate (95% CI)	Estimate (95% CI)	Estimate (95% CI)	Estimate (95% CI)	Estimate (95% CI)	Estimate (95% CI)	Estimate (95% CI)	Estimate (95% CI)
Model 1								
All	0.99 (0.87, 1.12)	1.11 (1.01, 1.20)	1.06 (0.94, 1.18)	1.14 (1.06, 1.23)	1.48 (1.40, 1.56)	1.65 (1.62, 1.67)	1.83 (1.80, 1.85)	1.96 (1.94, 1.99)
By gender								
Model 2a								
Males	0.87 (0.70, 1.05)	0.96 (0.83, 1.09)	0.79 (0.63, 0.95)	1.05 (0.94, 1.17)	1.10 (0.99, 1.21)	1.33 (1.29, 1.37)	1.50 (1.47, 1.54)	1.62 (1.58, 1.65)
Model 2b								
Females	1.10 (0.91, 1.28)	1.21 (1.08, 1.35)	1.31 (1.13, 1.48)	1.17 (1.03, 1.30)	1.67 (1.54, 1.79)	1.83 (1.79, 1.87)	1.96 (1.93, 2.00)	1.98 (1.95, 2.02)
By grade								
Model 3a								
Year 7	1.04 (0.79, 1.28)	1.21 (0.97, 1.44)	1.04 (0.81, 1.27)	1.17 (0.95, 1.38)	1.35 (1.15, 1.54)	1.58 (1.52, 1.64)	1.82 (1.76, 1.87)	1.97 (1.91, 2.03)
Model 3b								
Year 9	0.93 (0.69, 1.17)	0.98 (0.77, 1.19)	1.06 (0.85, 1.27)	1.11 (0.92, 1.31)	1.46 (1.26, 1.66)	1.53 (1.47, 1.59)	1.79 (1.74, 1.85)	1.99 (1.93, 2.05)
Model 3c								
Year 11	0.98 (0.76, 1.21)	1.16 (0.92, 1.39)	1.18 (0.97, 1.39)	1.18 (0.97, 1.38)	1.33 (1.12, 1.54)	1.52 (1.45, 1.59)	1.60 (1.54, 1.67)	1.85 (1.79, 1.91)
By family affluence								
Model 4a								
Low FAS	0.98 (0.75, 1.22)	1.18 (0.99, 1.37)	1.10 (0.86, 1.34)	1.23 (1.08, 1.38)	1.60 (1.43, 1.78)	1.78 (1.72, 1.84)	1.92 (1.87, 1.98)	2.04 (1.99, 2.09)
Model 4b								
Medium FAS	1.04 (0.85, 1.23)	1.14 (1.00, 1.29)	1.07 (0.89, 1.26)	1.09 (0.95, 1.22)	1.49 (1.37, 1.62)	1.65 (1.61, 1.69)	1.85 (1.81, 1.89)	1.93 (1.88, 1.97)
Model 4c								
High FAS	0.94 (0.69, 1.20)	0.97 (0.80, 1.14)	1.00 (0.81, 1.20)	1.11 (0.93, 1.29)	1.39 (1.25, 1.53)	1.58 (1.53, 1.62)	1.74 (1.69, 1.78)	1.95 (1.91, 1.99)

*Note*: All includes individuals across Years 7–11. Perceived schoolwork pressure was coded from 0 to 4, with a higher score denoting greater schoolwork pressure.

Abbreviation: FAS, Family Affluence Scale.

### Research question 4: To what extent are trends in emotional problems attenuated by adjustment for schoolwork pressure?

Linear regressions revealed that survey year remained a significant predictor of emotional problems after adjusting for perceived schoolwork pressure, however, estimated differences in mean emotional problem scores by survey year were reduced (Table 2). Adjusting for schoolwork pressure led to a reduction in the estimated cohort difference between 2009 and 2017 from 1.69 (95% CI = 1.58, 1.79) to 1.27 (95% CI = 1.16, 1.75), representing an overall reduction of 25%. A significant overall reduction was found when comparing cohort difference in emotional problems between 2009 and 2021, and a slightly larger overall reduction was found when comparing 2009–2019 (estimates reduced from 2.23 (95% CI = 2.12, 2.34) to 1.64 (95% CI = 1.54, 1.75), representing a reduction of 26% after adjusting for schoolwork pressure).

Further tests investigated the impact of adjusting for changes in schoolwork pressure in relation to emotional problem trends among males and females, younger and older students, and for each family affluence group separately (Table S10). When comparing cohort differences in emotional problems by gender: estimates reduced by around 19% for males after adjustment for schoolwork pressure, and by around 29% for females after adjusting for schoolwork pressure. Comparison of cohort different in emotional problems by age revealed a reduction in estimates of around 24% for those in Year 7, and by 14% among those in Year 11. For family affluence, estimates reduced by 26% among those in low affluent households, and by 23% and 26% among those from medium or high family affluence.

## DISCUSSION

In this study, we explored change in perceived schoolwork pressure among adolescents in Wales between 2002 and 2021, and associations with change in adolescent emotional problems over this period. We show that the proportion of young people in Wales perceiving high levels of schoolwork pressure increased between 2009 and 2021, mirroring similar increases in emotional problems. Prior to this, trends were largely stable or showed some improvement. Analyses also showed some attenuation of emotional problem trends when adjusting for perceived schoolwork pressure. Based on these findings, it is possible that changes in perceived schoolwork pressure may have accounted for some of the rise in emotional problems from 2009 onwards.

### The relationship between perceived schoolwork pressure and emotional problems

In line with previous research, our findings show an association between perceived school pressure and emotional problems. There are various possible explanations for this; school pressure may increase the risk of emotional problems; emotional problems may impact education and therefore academic pressure; perceptions of academic pressure may be symptomatic of emotional problems; or, confounding may explain the connection between the two. A recent systematic review emphasises the need for more high‐quality longitudinal evidence to distinguish these possibilities, but provides evidence that some longitudinal studies, which adjust for baseline mental health, show a prospective link between schoolwork pressure and subsequent mental health (Steare et al., 2023). Research on the Swedish educational reform supports similar conclusions. Within these reforms, more testing and stringent criteria for accessing further education were introduced, which increased overall school‐related stress in Sweden relative to other countries, and had an indirect effect on psychosomatic symptoms (Cashman et al., 2023; Högberg et al., 2019). Evidence has also shown that secular trends in youth emotional problems vary according to the proximity of mental health assessment to exams (West & Sweeting, 2003). Together these findings indicate that schoolwork pressure may act as a stressor that increases risk for emotional problems in young people, however, further longitudinal is needed to establish schoolwork pressure as a causal risk factor.

### Trends in perceived schoolwork pressure in Wales

The rise in perceived schoolwork pressure among adolescents in the present study was observed from 2009 onwards. This aligns with trends reported elsewhere (Cosma et al., 2022; De Looze et al., 2020; Högberg et al., 2020), and may be explained by an increase in the perceived importance of succeeding academically. Research has shown that as countries grow richer and more educated, school stress among adolescents also increases (Högberg, 2021). Schools classified as being higher in socioeconomic status are more likely to report increased schoolwork pressures (Brons et al., 2024). While our findings suggest minimal differences among groups based on family affluence, attending University is now the norm in many high‐income countries. This may prompt greater pressure to meet higher cultural expectations and academic demands. Notably, the trend for schoolwork pressure coincides with periods of major economic change, with perceived schoolwork pressure reducing during the early 2000s when investment in schools increased, and increasing following the economic crisis, a period which included substantial reductions in school spending (Sibieta, 2019). The increase in schoolwork pressures may therefore be a reflection of wider economic challenges as students face rising demands without the necessary resources to support them.

Increases in schoolwork pressure in the present study were evident among both males and females, but females perceived the most schoolwork pressure, aligning with much of the existing evidence (Cosma et al., 2020, 2022; Högberg et al., 2020). Explanations for these gender differences are complex and likely multifactorial. There is some evidence that females possess higher levels of conscientiousness than males, and these levels increase across development (Slobodskaya & Kornienko, 2021). It is possible that greater conscientiousness translates into greater school‐related pressures among female adolescents (Giota & Gustafsson, 2016). This could reflect the higher investment in education which generates more pressure among females as they try to avoid heightened repercussions from academic failures.

It is unclear from current findings whether changes in perceived schoolwork pressure reflect schools being more pressurised, whether young people's own expectations or wider societal expectations have changed, or whether young people have become less able to cope with pressures due to other challenges. Changes to perceived academic demands and schoolwork pressures are likely governed by many factors that differ across genders, including the biological and physiological experience of mental health problems (Bangasser & Cuarenta, 2021), differences in reporting and coping styles, as well as wider parental and societal expectations. Some have suggested that school‐related pressures may be increasingly heightened among females who must now balance the changing norms of female education and economic participation with efforts to maintain female identity and appearance during adolescence (Campbell et al., 2021). It is also possible that the introduction of more recent stresses, such as those on social media, place increasing pressures on females relative to males (Booker et al., 2018). However, debates surrounding causality prevent firm conclusions. Overall, further research is necessary to unpick possible reasons for gender differences in relation to schoolwork pressure, and to determine whether schoolwork pressures are increasing, or whether changes reflect wider cultural shifts.

### The role of increased schoolwork pressure in relation to emotional problems in Wales

The association between schoolwork pressure and emotional problems was shown to increase in strength over time in our study, mirroring previous research (Högberg, 2021). These findings suggest that schoolwork pressure has become more closely linked with emotional problems in adolescence. In support of this, we find that the estimated increase in emotional problems between 2009 and 2021 was attenuated when accounting for changes in perceived schoolwork pressure. This was the case for every demographic group but particularly so for females.

It is likely that the increasing burden of schoolwork pressure reflects wider cultural shifts, including greater academic demands and expectations from parents and wider society, as well as increasing costs of living and rising social inequalities. Together these may be driving increased pressure on young people to succeed academically.

### Implications

Our findings raise an important population health concern; adolescents in Wales report experiencing increased levels of pressure from their schoolwork, and these pressures are consistently associated with higher overall levels of emotional problems for young people today. While supporting adolescents to more effectively manage school‐related stress and exam anxiety could be important, our findings revealed increases in perceived schoolwork pressure between 2019 and 2021, a time in which many school assessments were cancelled due to the global pandemic. This suggests that increases in schoolwork pressure over time may not solely reflect increased assessments. It is perhaps relevant that prior research has documented long‐term changes in young people's personality traits such as increased perfectionism (Curran & Hill, 2019), greater individualism (Twenge, 2013), as well as more pessimistic views around post‐school opportunities and employment. Further research is needed to provide a more fine‐grained analysis of drivers.

Understanding why it is that young people experience high levels of pressure at school could inform potential interventions at the individual, school, or policy‐level. It is possible for example, that approaches to teaching and assessments may need to be re‐evaluated to ensure that practices are effective but also considerate of student wellbeing. While our findings suggest small variation between schools in levels of schoolwork pressure, which decreased across survey years, there may be a role, albeit small for school‐level influences on schoolwork pressure. The minimal role of schools could reflect similar pressures relating to exams and curricula across schools in Wales, which have likely become more similar over time due to the introduction of new standards and targets for all schools. Nevertheless, it remains possible that levels of teacher and classmate support, as well as school connectedness and climate, could play a small but significant role in reducing schoolwork pressures and subsequent problems. Indeed, whole‐school approaches that promote collaborative action across the school system appear promising in promoting the mental health of young people (Fazel et al., 2014).

Efforts to reduce and support adolescents with school‐related pressures will need to be accompanied by understanding of the broader cultural shifts. It is likely that a change in attitudes towards education, and a stronger appreciation of non‐academic skills and achievements, will be necessary to support young people with concerns about their education and future. While capturing social and cultural influences will be a challenge, it is paramount to understanding the cost of modern life on populational mental health (Eckersley, 2006). These efforts should focus in particular on older adolescents, who were shown to report consistently high schoolwork pressure and emotional problems within our study.

### Strengths and limitations

Our study benefits from using large, national‐based surveys with comparable data on perceived schoolwork pressure and emotional problems. A key limitation of our study, however, is that it was not possible to test the direction of association between schoolwork pressure and emotional problems. Both our measure of emotional problems and schoolwork pressure relied on self‐report, which could have inflated associations between perceived schoolwork pressure and emotional problems. Anotherissue relates to the possibility of rating shifts over time. In other words, young people in more recent surveys could be more willing to express their concerns, whether that be worries about schoolwork or mental health problems. However, several factors suggest this is an unlikely explanation for changes over time in schoolwork pressure and emotional problems. First, while young people have become more negative in their ratings of both emotional problems and schoolwork pressure, other evidence argues against the notion that young people are simply reporting more negatively across every aspect of their lives. For example, ratings of bullying in these surveys remained relatively stable over time (Anthony et al., 2023). Secondly, evidence now suggests that increasing mental health problems are largely specific to emotional problems, with no clear evidence that other mental health difficulties or neurodevelopmental conditions have increased over this time period (Sadler et al., 2018). This specificity makes it less likely that observed increases reflect a general shift in reporting. In addition, research has provided evidence that while attitudes towards mental health have changed over recent decades, a reduction in stigma about mental health does not consistently mirror change in self‐reported mental health problems (Gagné et al., 2023). Such findings provide evidence against the hypothesis that changes to attitudes and reporting may be driving the rise.

Other limitations to note are that there were some differences in methods across surveys, including the month of data collection, school coverage, and changes in delivery of the surveys from fieldworkers to teacher‐led. Students who reported that neither gender best described them in 2019 or 2021 were also excluded from analyses. Previous research in Wales have shown that these individuals have particularly high levels of emotional problems (Page et al., 2021), meaning current estimates of emotional problems may be conservative.

Finally, the study relied on a relatively crude indicator of perceived school pressure. While this has been used across the literature to enable cross‐country comparisons (Cosma et al., 2022), an important next step will be to better understand what it is that young people feel anxious about in relation to their school experience.

## CONCLUSIONS

Our study raises important population health concerns regarding schoolwork pressures and emotional problems of adolescents. Overall increases in schoolwork pressure may have contributed to the rise in adolescent emotional problems, particularly among female adolescents. Understanding reasons for the increasing school‐related pressures is necessary to preventing further increases and mental health problems. More broadly, a cultural shift towards the academic demands placed on young people will be necessary in supporting the healthy development of future generations.

## AUTHOR CONTRIBUTIONS

**Jessica M. Armitage**: Conceptualization; formal analysis; investigation; writing—original draft; writing—review and editing. **Gemma Lewis**: Conceptualization; writing—review and editing. **Nicholas Page**: Conceptualization; methodology; writing—review and editing. **Foteini Tseliou**: Writing—review and editing. **Rebecca Anthony**: Writing—review and editing. **Simon Murphy**: Writing—review and editing. **Graham Moore**: Writing—review and editing. **Stephan Collishaw**: Conceptualization; methodology; supervision; writing—review and editing.

## CONFLICT OF INTEREST STATEMENT

The authors declare no conflicts of interest.

## ETHICAL CONSIDERATIONS

Informed consent has been obtained. Students were provided with information about the surveys, and the first question asked for their consent to take part which if they declined closed the survey. Students also had the opportunity to withdraw from data collection at any time. Ethical approvals for surveys carried out in 2013, 2017, 2019, and 2021 were conducted by the Cardiff University School of Social Sciences Research Ethics Committee. Surveys prior to this were delivered by Welsh Government.

## Supporting information

Tables S1–S10

## Data Availability

Data from the Student Health and Well‐being (SHW) surveys are available for research purposes. Applicants will need to complete a data access request and re‐turn a signed data use protocol. Data requests will be reviewed, and a decision regarding data access made within 6 weeks. Provisioning of data will commence following confirmation of approval. Please contact SHRN at Cardiff University for further details (SHRN@cardiff.ac.uk).
