# Supplementary material for: Change over time in perceived schoolwork pressure and associations with emotional problems among 11–16‐year‐olds: A repeat cross‐sectional study in Wales, UK
Source: JCPP Adv. 2025 Mar 3;5(4):e70005. doi: 10.1002/jcv2.70005 (PMC12698275; doi:10.1002/jcv2.70005)
Supplement: Supplementary file 1 — Tables S1–S10 [file JCV2-5-e70005-s001.docx]

**Supporting Information**

**Change over time in perceived schoolwork pressure and associations with emotional problems among 11-16-year-olds: a repeat cross-sectional study in Wales, UK**

Jessica Armitage^1,2^, Gemma Lewis^3^, Nicholas Page^4^, Foteini Tseliou^1,2^, Rebecca Anthony^1,4^, Simon Murphy^1,4^, Graham Moore^1,4^, Stephan Collishaw^1,2^

^1^ Wolfson Centre for Young People’s Mental Health, Cardiff University, Cardiff, UK.

^2^ Division of Psychological Medicine and Clinical Neurosciences, MRC Centre for Neuropsychiatric Genetics and Genomics, Cardiff University School of Medicine, Cardiff University, Cardiff, UK.

^3^ Division of Psychiatry, Faculty of Brain Sciences, University College London, London, UK.

^4^ Centre for Development, Evaluation, Complexity and Implementation in Public Health Improvement (DECIPHer), School of Social Sciences, Cardiff University, Cardiff, UK.

| **Table S1:** Sample size and missing data across survey years | | | | | | | | |
| --- | --- | --- | --- | --- | --- | --- | --- | --- |
|  | **2002** | **2004** | **2006** | **2009** | **2013** | **2017** | **2019** | **2021** |
| Baseline | 4,104 | 7,057 | 4,409 | 9,194 | 9,055 | 103,971 | 119,388 | 123,204 |
| Complete school pressure, emotional problems, gender (any) and FAS, all students* | 3,442  (84%) | 6,156  (87%) | 3,977  (90%) | 7,544  (82%) | 8,432  (93%) | 86,845  (84%) | 100,588  (83%) | 101,570  (82%) |
| Complete school pressure, emotional problems, gender (male or female), and FAS, all students* | 3,442  (84%) | 6,156  (87%) | 3,977  (90%) | 7,544  (82%) | 8,432  (93%) | 85,795  (83%) | 99,128  (83%) | 98,088  (80%) |
| FAS = Family Affluence Scale  *Note: Survey years 2002 and 2006 did not collect data from students in Years 8 or 10. | | | | | | | | |

| **Table S2:** Demographics of complete and missing samples across survey years | | | | | | | | |
| --- | --- | --- | --- | --- | --- | --- | --- | --- |
|  | **2002** | **2004** | **2006** | **2009** | **2013** | **2017** | **2019** | **2021** |
| **Whole sample, N** | 4,104 | 7,057 | 4,409 | 9,194 | 9,055 | 103,971 | 119,388 | 123,204 |
| **Analysis sample, N**  (% complete) | 3,442  (84%) | 6,156  (87%) | 3,977  (90%) | 7,544  (82%) | 8,432  (93%) | 85,795  (83%) | 99,128  (83%) | 98,088  (80%) |
| **Sex**  **%** Females in whole sample  % Females in analysis sample | 48.3%  49.2% | 51.5%  52.4% | 50.7%  51.8% | 49.8%  51.7% | 49.4%  50.1% | 50.5%  50.7% | 50.2%  50.3% | 48.7%  49.0% |
| **Family Affluence**  **%** High family affluence in whole sample  % High family affluence in analysis sample | 26.1%  26.2% | 30.3%  30.4% | 31.5%  31.7% | 21.3%  21.3% | 29.8%  29.7% | 34.2%  34.7% | 34.6%  35.2% | 38.4%  39.5% |

| **Table S3:** Percentage of students aged 11-16 years who reported feeling a lot of schoolwork pressure between 2002 and 2021 in Wales | | | | | | | | |
| --- | --- | --- | --- | --- | --- | --- | --- | --- |
|  | **2002**  (n=3,442) | **2004**  (n=6,156) | **2006**  (n=3,977) | **2009**  (n=7,544) | **2013**  (n=8,432) | **2017**  (n=85,795) | **2019**  (n=99,128) | **2021**  (n=98,088) |
| All | 21.3%  (20.0, 22.7) | 23.8%  (22.8, 24.9) | 17.8%  (16.7, 19.0) | 13.2%  (12.5, 14.0) | 16.0%  (15.2, 16.8) | 23.8%  (23.5, 24.1) | 25.5%  (25.3, 25.8) | 25.9%  (25.7, 26.2) |
| Years 7, 9 and 11 | 21.3%  (20.0, 22.7) | 25.3%  (23.9, 27.0) | 17.8%  (16.7, 19.0) | 15.2%  (14.1, 16.3) | 15.5%  (14.5, 16.5) | 23.5%  (23.2, 23.9) | 25.1%  (24.8, 25.5) | 25.7%  (25.4, 26.1) |
| **Gender** | | | | | | | | |
| Males | 20.5%  (28.7, 22.5) | 23.2%  (21.7, 24.7) | 17.3%  (15.7, 19.1) | 11.7%  (10.7, 12.8) | 11.6%  10.6, 12.6) | 19.3%  (18.9, 19.7) | 19.9%  (19.6, 20.3) | 18.6%  (18.3, 19.0) |
| Females | 22.1%  (20.2, 24.1) | 24.4%  (22.9, 25.9) | 18.3%  (16.7, 20.0) | 14.6%  (13.6, 15.8) | 20.4%  (19.2, 21.6) | 28.1%  (27.7, 28.5) | 30.9%  (30.5, 31.3) | 33.5%  (33.1, 34.0) |
| **Age groups** | | | | | | | | |
| Year 7 | 11.4%  (9.7, 13.5) | 13.7%  (11.8, 15.9) | 9.5%  (8.0, 11.3) | 6.6%  (5.3, 8.0) | 5.2%  (4.2, 6.3) | 10.2%  (9.8, 10.7) | 11.2%  (10.8, 11.6) | 12.8%  (12.3, 13.2) |
| Year 8 | - | 18.1%  (16.1, 20.4) | - | 6.6%  (5.5, 7.9) | 7.5%  (6.3, 8.8) | 15.5%  (14.9, 16.0) | 16.4%  (15.9, 16.9) | 18.1%  (17.6, 18.6) |
| Year 9 | 12.4%  (20.7, 14.3) | 19.3%  (17.3, 21.5) | 11.0%  (9.5, 12.7) | 10.3%  (8.9, 11.8) | 11.4%  (10.0, 12.9) | 20.9%  (20.3, 21.5) | 22.7%  (22.1, 23.3) | 22.0%  (21.4, 22.6) |
| Year 10 | - | 25.5%  (23.2, 28.0) | - | 14.6%  (12.9, 16.5) | 26.5%  (24.4, 28.7) | 33.9%  (33.2, 34.7) | 36.8%  (36.1, 37.5) | 34.8%  (34.1, 35.5) |
| Year 11 | 41.2%  (38.3, 44.2) | 43.2%  (40.4, 46.1) | 33.4%  (30.8, 36.0) | 28.5%  (26.2, 30.8) | 31.9%  (29.6, 34.3) | 42.6%  (41.8, 43.4) | 44.3%  (43.6, 45.1) | 44.3%  (43.6, 45.0) |
| **Family affluence** | | | | | | | | |
| Low | 20.9%  (18.6, 23.5) | 25.2%  (23.2, 27.4) | 18.6%  (16.4, 21.0) | 13.1%  (11.9, 14.4) | 15.6%  (14.1, 17.2) | 23.7%  (23.1, 24.4) | 26.4%  (25.8, 27.0) | 26.5%  (26.0, 27.0) |
| Medium | 21.4%  (19.4, 23.5) | 23.9%  (22.3, 25.6) | 16.5%  (14.7, 18.3) | 13.0%  (11.9, 14.3) | 15.1%  (13.9, 16.2) | 23.1%  (22.7, 23.5) | 24.9%  (24.5, 25.3) | 25.2%  (24.7, 25.7) |
| High | 21.6%  (19.0, 24.4) | 22.4%  (20.6, 24.3) | 18.9%  (16.8, 21.1) | 13.7%  (12.1, 15.4) | 17.8%  (16.3, 19.3) | 25.0%  (24.3, 25.3) | 25.8%  (25.3, 26.2) | 26.1%  (25.7, 26.6) |

| **Table S4:** Percentage of students aged 11-16 years who reported feeling a lot of schoolwork pressure between 2002 and 2021 in Wales (presented by year group) | | | | | | | | |
| --- | --- | --- | --- | --- | --- | --- | --- | --- |
|  | **2002**  (n=3,442) | **2004**  (n=6,156) | **2006**  (n=3,977) | **2009**  (n=7,544) | **2013**  (n=8,432) | **2017**  (n=85,795) | **2019**  (n=99,128) | **2021**  (n=98,088) |
| **Age group by gender**  **Males** | | | | | | | | |
| Year 7 | 12.9%  (10.4, 16.0) | 15.8%  (13.0, 19.2) | 10.4%  (8.2, 13.2) | 7.5%  (5.7, 9.7) | 5.5%  (4.2, 7.3) | 11.2%  (10.6, 11.9) | 11.5%  (10.9, 12.2) | 12.0%  (11.4, 12.7) |
| Year 8 | - | 18.9%  (15.9, 22.2) | - | 6.9%  (5.3, 8.9) | 6.7%  (5.2, 8.5) | 14.2%  (13.5, 14.9) | 14.6%  (14.0, 15.3) | 14.2%  (13.5, 14.9) |
| Year 9 | 12.2%  (9.9, 15.0) | 18.9%  (15.9, 21.9) | 12.0%  (9.8, 14.6) | 9.7%  (7.8, 12.0) | 8.3%  (6.7, 10.2) | 16.9%  (16.2, 17.7) | 17.8%  (17.1, 18.6) | 15.0%  (14.4, 15.7) |
| Year 10 | - | 24.5%  (21.3, 28.0) | - | 12.6%  (10.4, 15.3) | 16.7%  (14.3, 19.4) | 25.8%  (24.9, 26.8) | 26.7%  (25.8, 27.6) | 22.8%  (22.0, 23.6) |
| Year 11 | 37.0%  (33.1, 41.1) | 38.8%  (34.7, 42.9) | 29.4%  (26.0, 33.1) | 21.7%  (18.9, 24.9) | 22.4%  (19.6, 25.5) | 31.2%  (30.1, 32.2) | 31.7%  (30.7, 32.7) | 31.0%  (30.1, 32.0) |
| **Age group by gender**  **Females** | | | | | | | | |
| Year 7 | 10.0%  (7.7, 12.7) | 11.8%  (9.4, 14.7) | 8.7%  (6.8, 11.2) | 5.7%  (4.2, 7.7) | 4.8%  (3.6, 6.5) | 9.2%  (8.6, 9.8) | 10.9%  (10.3, 11.5) | 13.5%  (12.9, 14.2) |
| Year 8 | - | 17.5%  (14.9, 20.6) | - | 6.3%  (4.9, 8.1) | 8.2%  (6.6, 10.3) | 16.7%  (15.9, 17.5) | 18.1%  (17.4, 18.9) | 22.2%  (21.4, 23.1) |
| Year 9 | 12.6%  (10.2, 15.5) | 19.8%  (17.1, 22.9) | 10.1%  (8.1, 12.5) | 10.8%  (8.9, 13.1) | 14.7%  (12.5, 17.2) | 24.7%  (23.8, 25.6) | 27.3%  (26.5, 28.2) | 29.3%  (28.4, 30.2) |
| Year 10 | - | 26.6%  (23.3, 30.2) | - | 16.3%  (13.9, 19.1) | 35.7%  (32.6, 39.0) | 41.4%  (40.4, 42.5) | 46.5%  (45.5, 47.5) | 47.4%  (46.3, 48.3) |
| Year 11 | 45.7%  (41.5, 50.0) | 47.2%  (43.3, 51.2) | 37.2%  (33.6, 41.0) | 35.1%  (31.8, 38.6) | 41.6%  (38.1, 45.1) | 53.3%  (52.2, 54.4) | 56.2%  (55.1, 57.2) | 57.4%  (56.4, 58.4) |
| **Age group by family affluence**  **Low** | | | | | | | | |
| Year 7 | 10.9%  (8.1, 14.7) | 13.5%  (10.0, 18.0) | 10.4%  (7.5, 14.1) | 7.9%  (5.9, 10.5) | 5.9%  (4.1, 8.4) | 12.5%  (11.5, 13.6) | 13.9%  (12.9, 14.9) | 14.6%  (13.7, 15.5) |
| Year 8 | - | 19.1%  (15.2, 23.7) | - | 8.0%  (6.1, 10.5) | 8.0%  (5.7, 10.9) | 16.1%  (15.0, 17.3) | 17.6%  (16.5, 18.7) | 19.4%  (18.4, 20.5) |
| Year 9 | 13.5%  (10.4, 17.3) | 22.5%  (18.5, 27.1) | 12.5%  (9.5, 16.2) | 8.4%  (6.4, 10.9) | 12.9%  (10.0, 16.4) | 20.1%  (18.8, 21.4) | 23.4%  (22.1, 24.7) | 22.7%  (21.7, 23.8) |
| Year 10 | - | 23.0%  (18.9, 27.6) | - | 12.9%  (10.4, 16.0) | 23.7%  (19.8, 28.2) | 32.6%  (31.1, 34.2) | 37.1%  (35.7, 38.6) | 34.4%  (33.2, 35.7) |
| Year 11 | 38.6%  (33.7, 43.8) | 46.0%  (40.9, 51.4) | 32.5%  (28.0, 37.5) | 28.9%  (25.2, 32.8) | 31.6%  (27.0, 36.7) | 40.8%  (39.1, 42.4) | 44.1%  (42.6, 45.7) | 43.1%  (41.8, 44.5) |
| **Medium family affluence** | | | | | | | | |
| Year 7 | 11.1%  (8.5, 14.3) | 13.1%  (10.3,16.5) | 9.3%  (7.1, 12.1) | 5.0%  (3.4, 7.2) | 4.7%  (3.4, 6.5) | 9.8%  (9.2, 10.5) | 10.9%  (10.2, 11.5) | 13.1%  (12.3, 14.0) |
| Year 8 | - | 17.6%  (14.6, 21.1) | - | 6.3%  (4.7, 8.4) | 6.7%  (5.2, 8.6) | 15.5%  (14.7, 16.3) | 16.0%  (15.2, 16.7) | 17.6%  (16.7, 18.5) |
| Year 9 | 11.3%  (8.8, 14.3) | 18.6%  (15.7, 22.0) | 9.9%  (7.7, 12.6) | 11.6%  (9.4, 14.2) | 9.9%  (8.1, 12.2) | 19.8%  (18.9, 20.7) | 21.6%  (20.8, 22.5) | 21.4%  (20.4, 22.4) |
| Year 10 | - | 27.6%  (23.8, 31.7) | - | 14.0%  (11.5, 16.9) | 27.3%  (24.2, 30.7) | 32.8%  (31.7, 33.8) | 36.6%  (35.5, 37.6) | 34.0%  (32.9, 35.2) |
| Year 11 | 40.9%  (36.7, 45.3) | 41.5%  (37.4, 45.7) | 30.5%  (26.7, 34.6) | 28.2%  (24.8, 31.9) | 28.6%  (25.4, 32.0) | 41.2%  (40.0, 42.4) | 43.5%  (42.4, 44.6) | 43.3  (42.0, 44.7) |
| **High family affluence** | | | | | | | | |
| Year 7 | 12.5%  (9.2, 16.8) | 14.7%  (11.4, 18.8) | 9.1%  (6.7, 12.4) | 6.9%  (4.5, 10.6) | 5.2%  (3.6, 7.5) | 9.2%  (8.5, 10.0) | 9.8%  (9.1, 10.5) | 11.1%  (10.4, 11.8) |
| Year 8 | - | 18.1%  (14.7, 22.0) | - | 4.9%  (3.1, 7.7) | 8.3%  (6.1, 11.0) | 15.1%  (14.2, 16.0) | 16.3%  (15.5, 17.1) | 17.5%  (16.7, 18.4) |
| Year 9 | 12.9%  (9.8, 16.8) | 17.4%  (14.1, 21.3) | 11.2%  (8.6, 14.4) | 10.8%  (8.0, 14.4) | 12.5%  (9.9, 15.5) | 22.6%  (21.7, 23.7) | 23.6%  (22.7, 24.6) | 22.0%  (21.0, 22.9) |
| Year 10 | - | 25.3%  (21.2, 29.8) | - | 19.1%  (15.0, 23.9) | 27.3%  (23.7, 31.2) | 36.2%  (35.0, 37.4) | 36.9%  (35.8, 38.1) | 35.7%  (34.6, 36.7) |
| Year 11 | 45.6%  (39.4, 52.0) | 43.0%  (37.3, 49.0) | 38.0%  (33.3, 42.9) | 28.2%  (23.5, 33.5) | 37.8%  (33.3, 42.4) | 45.7%  (44.3, 47.1) | 45.4%  (44.2, 46.7) | 45.9%  (44.7, 47.0) |

| **Table S5:** Coefficient estimates for change over time in schoolwork pressure among students in Wales between 2009 and 2021, interactions by gender and family affluence | | | | | | | | |
| --- | --- | --- | --- | --- | --- | --- | --- | --- |
|  | Main effect of cohort (unadjusted) | | Main effect of cohort (adjusted for gender, age, family affluence) | | Main effect of cohort (adjusted for gender, age, family affluence, gender by cohort interaction) | | Main effect of cohort (adjusted for gender, age, family affluence, family affluence by cohort interaction) | |
|  | Estimate (95% CI) | P value | Estimate  (95% CI) | P value | Estimate  (95% CI) | P value | Estimate  (95% CI) | P value |
| **2009** (ref) | | | | | | | | |
| **2013** (n=15,976) |  |  |  |  |  |  |  |  |
| Cohort | 0.04  (0.00, 0.08) | 0.017 | 0.06  (0.02, 0.09) | <0.001 | -0.01  (-0.05, 0.03) | 0.647 | 0.05  (0.00, 0.11) | 0.05 |
| Gender | - | - | 0.17  (0.15, 0.20) | <0.001 | 0.10  (0.06, 0.14) | <0.001 | 0.17  (0.15, 0.20) | <0.001 |
| Age | - | - | 0.22  (0.21, 0.23) | <0.001 | 0.22  (0.21, 0.23) | <0.001 | 0.22  (0.21, 0.23) | <0.001 |
| FAS | - | - | 0.02  (-0.00, 0.04) | 0.073 | 0.02  (-0.00, 0.04) | 0.065 | 0.00  (-0.02, 0.04) | 0.596 |
| Cohort x Gender  (male ref) | - | - | - | - | 0.13  (0.08, 0.19) | <0.001 | - | - |
| Cohort x medium family affluence  (low FAS ref) | - | - | - | - | - | - | -0.01  (-0.08, 0.05) | 0.722 |
| Cohort x high family affluence  (low FAS ref) | - | - | - | - | - | - | 0.04  (-0.04, 0.11) | 0.354 |
| **2017** (n=93,339) | | | | | | | | |
| Cohort | 0.26  (0.23, 0.28) | <0.001 | 0.28  (0.26, 0.31) | <0.001 | 0.23  (0.19, 0.26) | <0.001 | 0.30  (0.26, 0.34) | <0.001 |
| Gender | - | - | 0.22  (0.20, 0.23) | <0.001 | 0.11  (0.07, 0.15) | <0.001 | 0.22  (0.20, 0.23) | <0.001 |
| Age | - | - | 0.23  (0.23, 0.24) | <0.001 | 0.23  (0.23, 0.24) | <0.001 | 0.23  (0.23, 0.24) | <0.001 |
| FAS | - | - | 0.000  (-0.00,0.01) | 0.808 | 0.000  (-0.00, 0.00) | 0.776 | 0.00  (-0.02, 0.04) | 0.488 |
| Cohort x Gender | - | - | - | - | 0.12  (0.07, 0.16) | <0.001 | - | - |
| Cohort x medium family affluence  (low FAS ref) | - | - | - | - | - | - | -0.03  (-0.08, 0.02) | 0.268 |
| Cohort x high family affluence  (low FAS ref) | - | - | - | - | - | - | -0.02  (-0.09, 0.04) | 0.440 |
| **2019** (n=106,672) | | | | | | | | |
| Cohort | 0.33  (0.30, 0.35) | <0.001 | 0.36  (0.34, 0.39) | <0.001 | 0.28  (0.24, 0.31) | <0.001 | 0.41  (0.37, 0.45) | <0.001 |
| Gender | - | - | 0.26  (0.25, 0.28) | <0.001 | 0.11  (0.07, 0.15) | <0.001 | 0.26  (0.25, 0.28) | <0.001 |
| Age | - | - | 0.23  (0.23, 0.24) | <0.001 | 0.23  (0.22, 0.23) | <0.001 | 0.23  (0.22, 0.23) | <0.001 |
| FAS | - | - | -0.03  (-0.04, -0.02) | <0.001 | -0.03  (-0.04, -0.02) | <0.001 | 0.01  (-0.02, 0.04) | 0.366 |
| Cohort x Gender | - | - | - | - | 0.17  (0.12, 0.21) | <0.001 | - | - |
| Cohort x medium family affluence  (low FAS ref) | - | - | - | - | - | - | -0.06  (-0.12, -0.01) | 0.015 |
| Cohort x high family affluence  (low FAS ref) | - | - | - | - | - | - | -0.09  (-0.15, -0.03) | <0.01 |
| **2021** (n=105,057) | | | | | | | | |
| Cohort | 0.35  (0.33, 0.38) | <0.001 | 0.38  (0.36, 0.41) | <0.001 | 0.25  (0.22, 0.28) | <0.001 | 0.42  (0.38, 0.45) | <0.001 |
| Gender | - | - | 0.35  (0.33, 0.36) | <0.001 | 0.11  (0.06, 0.15) | <0.001 | 0.35  (0.33, 0.36) | <0.001 |
| Age | - | - | 0.20  (0.19, 0.20) | <0.001 | 0.20  (0.19, 0.20) | <0.001 | 0.20  (0.19, 0.20) | <0.001 |
| FAS | - | - | -0.02  (-0.02, -0.01) | <0.001 | -0.02  (-0.02, -0.01) | <0.001 | 0.01  (-0.01, 0.04) | 0.371 |
| Cohort x Gender | - | - | - | - | 0.26  (0.21, 0.30) | <0.001 | - | - |
| Cohort x medium family affluence  (low FAS ref) | - | - | - | - | - | - | -0.05  (-0.09, 0.00) | 0.057 |
| Cohort x high family affluence  (low FAS ref) | - | - | - | - | - | - | -0.06  (-0.12, 0.00) | 0.035 |
| Note: Estimates use 2009 as a reference. All models adjust for random effects of school. | | | | | | | | |

| **Table S6:** Between-school variance in schoolwork pressure across survey years | |
| --- | --- |
| Year | Between-school variance |
| 2002 | 0.192 |
| 2004 | 0.154 |
| 2006 | 0.120 |
| 2009 | 0.068 |
| 2013 | 0.034 |
| 2017 | 0.020 |
| 2019 | 0.020 |
| 2021 | 0.024 |

| **Table S7**: Mean emotional problem scores of students in Wales between 2002 and 2021 | | | | | | | | | |
| --- | --- | --- | --- | --- | --- | --- | --- | --- | --- |
|  | **2002**  (n=3,442) | **2004**  (n=6,156) | **2006**  (n=3,977) | **2009**  (n=7,544) | **2013**  (n=8,432) | **2017**  (n=85,795) | **2019**  (n=99,128) | **2021**  (n=98,088) |  |
| All | 4.74  (4.61, 4.86) | 5.03  (4.93, 5.12) | 4.52  (4.40, 4.64) | 4.13  (4.04, 4.21) | 4.64  (4.55, 4.73) | 5.70  (5.67, 5.72) | 6.10  (6.07, 6.13) | 6.76  (6.74, 6.79) |  |
| **Gender** | | | | | | | | |  |
| Males | 4.20  (4.03, 4.37) | 4.47  (4.34, 4.60) | 4.06  (3.91, 4.22) | 3.57  (3.46, 3.68) | 3.86  (3.75, 3.97) | 4.98  (4.94, 5.01) | 5.18  (5.14, 5.22) | 5.54  (5.50, 5.57) |  |
| Females | 5.29  (5.10, 5.47) | 5.53  (5.40, 5.67) | 4.95  (4.77, 5.12) | 4.64  (4.52, 4.77) | 5.42  (5.29, 5.55) | 6.38  (6.34, 6.42) | 6.98  (6.94, 7.02) | 8.04  (8.00, 8.08) |  |
| **Age groups** | | | | | | | | |  |
| Year 7 | 4.24  (4.02, 4.47) | 4.50  (4.28, 4.73) | 4.20  (3.99, 4.41) | 3.55  (3.36, 3.75) | 3.64  (3.46, 3.81) | 4.59  (4.53, 4.65) | 4.92  (4.87, 4.98) | 5.95  (5.89, 6.02) |  |
| Year 8 | - | 4.90  (4.69, 5.11) | - | 3.67  (3.49, 3.85) | 4.04  (3.87, 4.22) | 5.28  (5.22, 5.34) | 5.76  (5.70, 5.82) | 6.44  (6.38, 6.50) |  |
| Year 9 | 4.72  (4.50, 4.94) | 5.00  (4.79, 5.20) | 4.48  (4.29, 4.68) | 4.18  (4.00, 4.36) | 4.67  (4.48, 4.87) | 5.73  (5.67, 5.79) | 6.13  (6.07, 6.19) | 6.74  (6.67, 6.80) |  |
| Year 10 | - | 5.15  (4.94, 5.37) | - | 4.50  (4.31, 4.69) | 5.33  (5.13, 5.54) | 6.32  (6.25, 6.39) | 6.82  (6.75, 6.88) | 7.20  (7.14, 7.27) |  |
| Year 11 | 5.24  (5.03, 5.46) | 5.56  (5.34, 5.78) | 4.87  (4.66, 5.07) | 4.71  (4.51, 4.91) | 5.66  (5.44, 5.88) | 6.77  (6.70, 6.84) | 7.06  (6.99, 7.12) | 7.56  (7.50, 7.63) |  |
| **Family affluence** | | | | | | | | | |
| Low | 4.91  (4.68, 5.15) | 5.44  (5.25, 5.64) | 4.85  (4.61, 5.09) | 4.39  (4.24, 4.53) | 4.88  (4.69, 5.06) | 6.23  (6.19, 6.32) | 6.79  (6.73, 6.85) | 7.23  (7.17, 7.29) |  |
| Medium | 4.63  (4.44, 4.82) | 4.87  (4.72, 5.01) | 4.48  (4.30, 4.66) | 4.01  (3.88, 4.14) | 4.71  (4.57, 4.84) | 5.69  (5.65, 5.73) | 6.12  (6.08, 6.16) | 6.75  (6.70, 6.80) |  |
| High | 4.69  (4.44, 4.95) | 4.87  (4.70, 5.04) | 4.29  (4.09, 4.48) | 3.90  (3.73, 4.08) | 4.35  (4.20, 4.50) | 5.36  (5.31, 5.41) | 5.64  (5.60, 5.69) | 6.44  (6.40, 6.49) |  |

| **Table S8:** Coefficient estimates for change over time in emotional problems among students in Wales between 2009 and 2021, interactions by gender and family affluence | | | | | | | | |
| --- | --- | --- | --- | --- | --- | --- | --- | --- |
|  | Main effect of cohort (unadjusted) | | Main effect of cohort (adjusted for gender, age, and family affluence) | | Main effect of cohort (adjusted for gender, age, family affluence, and gender by cohort interaction) | | Main effect of cohort (adjusted for gender, age, family affluence, and family affluence by cohort interaction) | |
|  | Estimate (95% CI) | P value | Estimate  (95% CI) | P value | Estimate  (95% CI) | P value | Estimate  (95% CI) | P value |
| **2013** (n=15,976) | | | | | | | | |
| Cohort | 0.51  (0.37, 0.66) | <0.001 | 0.62  (0.47, 0.76) | <0.001 | 0.38  (0.20, 0.58) | <0.001 | 0.52  (0.29, 0.76) | <0.001 |
| Gender | - | - | 1.31  (1.19, 1.43) | <0.001 | 1.08  (0.90, 1.25) | <0.001 | 1.31  (1.19, 1.43) | <0.001 |
| Grade | - | - | 0.44  (0.39, 0.48) | <0.001 | 0.44  (0.39, 0.48) | <0.001 | 0.44  (0.39, 0.48) | <0.001 |
| FAS | - | - | -0.27  (-0.35, -0.19) | <0.001 | -0.27  (-0.35, -0.19) | <0.001 | -0.25  (-0.37, -0.13) | <0.001 |
| Cohort x Gender  (male ref) | - | - | - | - | 0.45  (0.21, 0.69) | <0.001 | - | - |
| Cohort x medium family affluence  (low FAS ref) | - | - | - | - | - | - | 0.23  (-0.05, 0.51) | 0.113 |
| Cohort x high family affluence  (low FAS ref) | - | - | - | - | - | - | -0.04  (-0.37, 0.29) | 0.815 |
| **2017** (n=93,339) | | | | | | | | |
| Cohort | 1.49  (1.38, 1.60) | <0.001 | 1.69  (1.58, 1.79) | <0.001 | 1.54  (1.40, 1.69) | <0.001 | 1.83  (1.66, 2.00) | <0.001 |
| Gender | - | - | 1.35  (1.30, 1.40) | <0.001 | 1.09  (0.91, 1.28) | <0.001 | 1.35  (1.29, 1.40) | <0.001 |
| Grade | - | - | 0.52  (0.50, 0.54) | <0.001 | 0.52  (0.50, 0.54) | <0.001 | 0.52  (0.50, 0.54) | <0.001 |
| FAS | - | - | -0.42  (-0.46, -0.39) | <0.001 | -0.42  (-0.46, -0.38) | <0.001 | -0.26  (-0.39, -0.13) | <0.001 |
| Cohort x Gender | - | - | - | - | 0.29  (0.09, 0.48) | <0.01 | - | - |
| Cohort x medium family affluence  (low FAS ref) | - | - | - | - | - | - | -0.13  (-0.35, 0.09) | 0.255 |
| Cohort x high family affluence  (low FAS ref) | - | - | - | - | - | - | -0.37  (-0.64, -0.10) | <0.01 |
| **2019** (n=106,672) | | | | | | | | |
| Cohort | 2.01  (1.89, 2.12) | <0.001 | 2.23  (2.12, 2.34) | <0.001 | 1.88  (1.73, 2.03) | <0.001 | 2.47  (2.30, 2.65) | <0.001 |
| Gender | - | - | 1.72  (1.67, 1.77) | <0.001 | 1.08  (0.89, 1.28) | <0.001 | 1.72  (1.67, 1.77) | <0.001 |
| Grade | - | - | 0.52  (0.50, 0.54) | <0.001 | 0.52  (0.50, 0.54) | <0.001 | 0.52  (0.50, 0.54) | <0.001 |
| FAS | - | - | -0.54  (-0.58, -0.51) | <0.001 | -0.54  (-0.58, -0.51) | <0.001 | -0.26  (-0.40, -0.13) | <0.001 |
| Cohort x Gender | - | - | - | - | 0.68  (0.48, 0.89) | <0.001 | - | - |
| Cohort x medium family affluence  (low FAS ref) | - | - | - | - | - | - | -0.23  (-0.46, -0.00) | 0.045 |
| Cohort x high family affluence  (low FAS ref) | - | - | - | - | - | - | -0.62  (-0.89, -0.34) | <0.001 |
| **2021** (n=105,632) | | | | | | | | |
| Cohort | 2.61  (2.49, 2.73) | <0.001 | 2.79  (2.68, 2.90) | <0.001 | 2.06  (1.91, 2.21) | <0.001 | 2.83  (2.66, 3.00) | <0.001 |
| Gender | - | - | 2.39  (2.34, 2.45) | <0.001 | 1.08  (0.88, 1.27) | <0.001 | 2.39  (2.34, 2.44) | <0.001 |
| Grade | - | - | 0.38  (0.37, 0.40) | <0.001 | 0.38  (0.37, 0.40) | <0.001 | 0.38  (0.37, 0.40) | <0.001 |
| FAS | - | - | -0.37  (-0.40, -0.33) | <0.001 | -0.36  (-0.40, -0.33) | <0.001 | -0.28  (-0.41, -0.14) | <0.001 |
| Cohort x Gender | - | - | - | - | 1.42  (1.21, 1.62) | <0.001 | - | - |
| Cohort x medium family affluence  (low FAS ref) | - | - | - | - | - | - | -0.02  (-0.25, 0.22) | 0.896 |
| Cohort x high family affluence  (low FAS ref) | - | - | - | - | - | - | -0.19  (-0.47, 0.08) | 0.166 |
| Note: Estimates use 2009 as a reference. All models adjust for random effects of school. | | | | | | | | |

| **Table S9:** Multi-level model of individual and school-level effects in analyses predicting emotional problem scores from schoolwork pressure | | | | |
| --- | --- | --- | --- | --- |
|  | **2013**  (n=8,432) | **2017**  (n=86,845) | **2019**  (n=100,588) | **2021**  (n=101,570) |
|  | Estimate  (95% CI) | Estimate  (95% CI) | Estimate  (95% CI) | Estimate  (95% CI) |
| Unadjusted model | 1.48 (1.40, 1.56)*** | 1.66 (1.63, 1.68)*** | 1.84 (1.82, 1.87)*** | 2.03 (2.01, 2.06)*** |
| Multi-level model adjusted for random school effects | 1.47 (1.38, 1.55)*** | 1.65 (1.63, 1.68) *** | 1.85 (1.83, 1.88) *** | 2.04 (2.02, 2.07) *** |
| Between-school variance | 0.25 | 0.20 | 0.21 | 0.18 |
| Within-school between-student variance | 14.49 | 15.75 | 16.55 | 16.99 |
| Total variance | 14.74 | 15.95 | 16.75 | 17.17 |
| VPC | 0.016 | 0.013 | 0.013 | 0.011 |
| Note: VPC=variance partition coefficient  The VPC indicates the variance in schoolwork pressure can be attributed to differences between schools. | | | | |

| **Table S10:** Coefficient estimates for change over time in emotional problems among students in Wales between 2009 and 2021, accounting for schoolwork pressure (by gender, age, and family affulence) | | | | | | | |
| --- | --- | --- | --- | --- | --- | --- | --- |
|  |  | Main effect of cohort (unadjusted) | | Main effect of cohort (adjusted for gender, age and family affluence)Ϯ | | Main effect of cohort (adjusted for gender, age, family affluence, and schoolwork pressure)Ϯ | |
|  | N | Estimate (95% CI) | P value | Estimate  (95% CI) | P value | Estimate  (95% CI) | P value |
| ***By gender*** | | | | | | | |
| **Males** |  |  |  |  |  |  |  |
| 2009 (ref) |  |  |  |  |  |  |  |
| 2013 | 7,852 | 0.30  (0.13, 0.44) | <0.001 | 0.37  (0.20, 0.54) | <0.001 | 0.38  (0.22, 0.55) | <0.001 |
| 2017 | 45,408 | 1.36  (1.22, 1.51) | <0.001 | 1.53  (1.38, 1.67) | <0.001 | 1.25  (1.11, 1.39) | <0.001 |
| 2019 | 52,199 | 1.63  (1.48, 1.78) | <0.001 | 1.84  (1.69, 1.99) | <0.001 | 1.46  (1.32, 1.60) | <0.001 |
| 2021 | 53,667 | 1.95  (1.80, 2.11) | <0.001 | 2.10  (1.95, 2.25) | <0.001 | 1.70  (1.56, 1.84) | <0.001 |
| **Females** |  |  |  |  |  |  |  |
| 2013 | 8,124 | 0.75  (0.53, 0.96) | <0.001 | 0.84  (0.63, 1.05) | <0.001 | 0.69  (0.49, 0.89) | <0.001 |
| 2017 | 47, 931 | 1.63  (1.47, 1.79) | <0.001 | 1.84  (1.68, 1.99) | <0.001 | 1.27  (1.18, 1.42) | <0.001 |
| 2019 | 54, 473 | 2.37  (2.20, 2.53) | <0.001 | 2.60  (2.44, 2.76) | <0.001 | 1.79  (1.64, 1.94) | <0.001 |
| 2021 | 51, 686 | 3.35  (3.19, 3.51) | <0.001 | 3.46  (3.30, 3.62) | <0.001 | 2.51  (2.36, 2.65) | <0.001 |
| **Age** |  |  |  |  |  |  |  |
| **Year 7** |  |  |  |  |  |  |  |
| 2013 | 3,062 | 0.06  (-0.22, 0.33) | 0.688 | 0.09  (-0.18, 0.37) | 0.506 | 0.18  (-0.08, 0.44) | 0.179 |
| 2017 | 18,977 | 0.99  (0.76, 1.23) | <0.001 | 1.11  (0.87, 1.34) | <0.001 | 0.87  (0.65, 1.09) | <0.001 |
| 2019 | 22,129 | 1.39  (1.15, 1.64) | <0.001 | 1.55  (1.31, 1.79) | <0.001 | 1.17  (0.95, 1.39) | <0.001 |
| 2021 | 20,826 | 2.40  (2.14, 2.65) | <0.001 | 2.51  (2.26, 2.76) | <0.001 | 1.91  (1.68, 2.14) | <0.001 |
| **Year 9** |  |  |  |  |  |  |  |
| 2013 | 3,408 | 0.47  (0.18, 0.76) | <0.001 | 0.54  (0.26, 0.83) | <0.001 | 0.52  (0.25, 0.79) | <0.001 |
| 2017 | 20,046 | 1.50  (1.27, 1.73) | <0.001 | 1.63  (1.41, 1.86) | <0.001 | 1.23  (1.02, 1.44) | <0.001 |
| 2019 | 22,338 | 2.05  (1.81, 2.29) | <0.001 | 2.17  (1.94, 2.41) | <0.001 | 1.58  (1.37, 1.80) | <0.001 |
| 2021 | 22,846 | 2.57  (2.33, 2.81) | <0.001 | 2.73  (2.51, 2.96) | <0.001 | 2.07  (1.86, 2.29) | <0.001 |
| **Year 11** |  |  |  |  |  |  |  |
| 2013 | 2,991 | 1.01  (0.67, 1.35) | <0.001 | 1.07  (0.74, 1.40) | <0.001 | 1.06  (0.74, 1.38) | <0.001 |
| 2017 | 16,350 | 2.08  (1.83, 2.34) | <0.001 | 2.18  (1.94, 2.43) | <0.001 | 1.92  (1.68, 2.15) | <0.001 |
| 2019 | 19,113 | 2.33  (2.08, 2.58) | <0.001 | 2.46  (2.22, 2.71) | <0.001 | 2.10  (1.87, 2.33) | <0.001 |
| 2021 | 19,302 | 2.90  (2.64, 3.15) | <0.001 | 2.98  (2.74, 3.23) | <0.001 | 2.57  (2.34, 2.80) | <0.001 |
| ***By family affluence*** | | | | | | | |
| **Low** | | | | | | | |
| 2013 | 4,857 | 0.49  (0.26, 0.72) | <0.001 | 0.52  (0.26, 0.78) | <0.001 | 0.55  (0.30, 0.80) | <0.001 |
| 2017 | 20,975 | 1.85  (1.65, 2.04) | <0.001 | 1.87  (1.68, 2.05) | <0.001 | 1.41  (1.24, 1.58) | <0.001 |
| 2019 | 24,208 | 2.41  (2.22, 2.61) | <0.001 | 2.46  (2.27, 2.65) | <0.001 | 1.79  (1.62, 1.97) | <0.001 |
| 2021 | 30,492 | 2.83  (2.64, 3.02) | <0.001 | 2.87  (2.69, 3.06) | <0.001 | 2.14  (1.97, 2.31) | <0.001 |
| **Medium** |  |  |  |  |  |  |  |
| 2013 | 7,002 | 0.77  (0.46, 0.88) | <0.001 | 0.74  (0.54, 0.94) | <0.001 | 0.69  (0.50, 0.88) | <0.001 |
| 2017 | 41,022 | 1.63  (1.46, 1.80) | <0.001 | 1.71  (1.55, 1.88) | <0.001 | 1.31  (1.16, 1.46) | <0.001 |
| 2019 | 45,921 | 2.16  (1.99, 2.34) | <0.001 | 2.23  (2.07, 2.40) | <0.001 | 1.67  (1.51, 1.82) | <0.001 |
| 2021 | 34,767 | 2.74  (2.56, 2.91) | <0.001 | 2.84  (2.67, 3.01) | <0.001 | 2.22  (2.06, 2.38) | <0.001 |
| **High** |  |  |  |  |  |  |  |
| 2013 | 4,117 | 0.45  (0.21, 0.69) | <0.001 | 0.50  (0.27, 0.74) | <0.001 | 0.40  (0.18, 0.63) | <0.001 |
| 2017 | 31,342 | 1.42  (1.20, 1.64) | <0.001 | 1.50  (1.29, 1.72) | <0.001 | 1.10  (0.90, 1.30) | <0.001 |
| 2019 | 36,543 | 1.76  (1.53, 1.99) | <0.001 | 1.83  (1.61, 2.05) | <0.001 | 1.34  (1.13, 1.55) | <0.001 |
| 2021 | 40,373 | 2.52  (2.28, 2.75) | <0.001 | 2.63  (2.41, 2.86) | <0.001 | 2.02  (1.81, 2.23) | <0.001 |
| Note: Estimates use 2009 as a reference. All models adjust for random effects of school.  Ϯ Covariates depend on variable that is stratified. Analyses stratified by gender adjust for age and family affluence, while analyses stratified by age adjust for gender and family affluence, and analyses stratified by family affluence adjust for gender and age. | | | | | | | |
